# Towards a Cure for HARS Disease

**DOI:** 10.3390/genes14020254

**Published:** 2023-01-18

**Authors:** Sarah D. P. Wilhelm, Rosan Kenana, Yi Qiu, Patrick O’Donoghue, Ilka U. Heinemann

**Affiliations:** 1 Department of Biochemistry, The University of Western Ontario, London, ON N6A 5C1, Canada; 2 Department of Chemistry, The University of Western Ontario, London, ON N6A 5C1, Canada

**Keywords:** aminoacyl-tRNA synthetase, genetic disease, neurodegeneration, mistranslation

## Abstract

Histidyl-tRNA synthetase (HARS) ligates histidine to its cognate transfer RNA (tRNA^His^). Mutations in HARS cause the human genetic disorders Usher syndrome type 3B (USH3B) and Charcot-Marie-Tooth syndrome type 2W (CMT2W). Treatment for these diseases remains symptomatic, and no disease specific treatments are currently available. Mutations in *HARS* can lead to destabilization of the enzyme, reduced aminoacylation, and decreased histidine incorporation into the proteome. Other mutations lead to a toxic gain-of-function and mistranslation of non-cognate amino acids in response to histidine codons, which can be rescued by histidine supplementation in vitro. We discuss recent advances in characterizing *HARS* mutations and potential applications of amino acid and tRNA therapy for future gene and allele specific therapy.

## 1. Aminoacyl-tRNA Synthetases in Translation

During translation, genetic information carried by each messenger RNA (mRNA) is used as a template to synthesize the corresponding sequence of amino acids of a particular protein. Protein synthesis can be divided into three steps: chain initiation, elongation, and termination. In translation initiation, mRNA is targeted by the translation machinery, which involves small (40S) and large (60S) ribosomal subunits, methionyl transfer RNA (Met-tRNA) decoding of the typical AUG start codon, and eukaryotic initiation factors (eIF) [1]. In chain elongation, an mRNA codon is paired with an anticodon of an aminoacyl-tRNA bound to the elongation factor, resulting in the transfer of one amino acid at a time from the tRNA molecule to the nascent chain of amino acids. The ribosome moves down the mRNA in the 5′ to 3′ direction until it reaches one of the three terminator codons (UAA, UAG, or UGA) resulting in the binding of a release factor, chain termination, and ribosome disassembly. The completed polypeptide is then released from the ribosome [2,3].

The fidelity of the genetic code is determined by the accuracy of these steps, and most significantly, by the accuracy of charging the transfer RNAs (tRNAs) with their cognate amino acid. In eukaryotes, there is a specific aminoacyl-tRNA synthetase (aaRS) for each of the 20 standard amino acids. The aaRSs belong to two independently evolved enzyme families, called class I and class II aaRSs [4]. Class I synthetases are usually monomeric with a Rossman fold including α-helices connecting a five-stranded parallel β sheet, whereas class II synthetases are usually dimeric or multimeric with a seven-stranded anti-parallel β-sheet fold flanked by α-helices. Within each class, subclasses are further divided to some extent according to their capacity to recognize chemically related amino acids [5].

**Figure 1:**
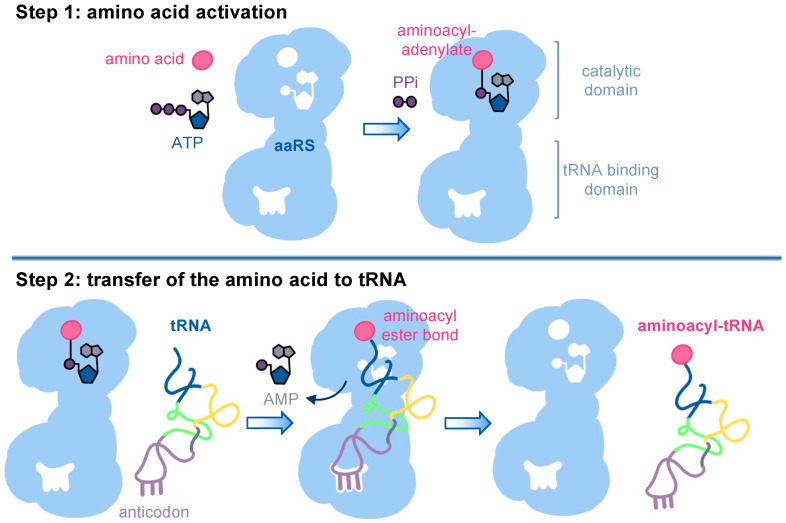
Aminoacylation of tRNAs occurs in a two-step process. Step 1: In the catalytic domain of the aminoacyl-tRNA synthetase (aaRS), a reaction between an amino acid and an ATP molecule results in an aminoacyl-AMP intermediate and releases a pyrophosphate (PPi). Step 2: tRNA recognition is aaRS-specific and involves binding of the acceptor stem, as well as tRNA identity elements such as the anticodon loop, variable loop, or other identity elements in the tRNA. In the aaRS catalytic domain, the amino acid is transferred from the aminoacyl-AMP intermediate to the cognate tRNA molecule. The aminoacyl-tRNA product is released from the enzyme.

Aminoacylation of tRNAs occurs in two steps (Figure 1). In the catalytic domain, adenosine triphosphate (ATP) and the cognate amino acid are bound, a high-energy phosphoanhydride bond in ATP is hydrolyzed to form aminoacyl adenylate-AMP, and inorganic pyrophosphate (PPi) is released. Next, the cognate tRNA is recruited to the aaRS, and the amino acid is transferred to the 3′ end of the tRNA at the acceptor stem, forming an aminoacyl-ester bond [6]. The aminoacyl-tRNAs then bind to the elongation factor and are competent substrates for protein synthesis on the ribosome.

All aaRSs contain a catalytic domain. aaRS amino acid specificity is determined by the composition and size of the amino acid binding pocket, and binding of the tRNA acceptor stem in the aaRS catalytic domain is a prerequisite to aminoacylation. Most aaRSs also contain a tRNA binding domain for the recognition of the tRNA anticodon loop. tRNA recognition relies on tRNA-specific recognition element nucleotides that are called identity elements. While many aaRSs recognize the anticodon as the primary identity element, bases in the acceptor stem and the variable loop are also common identity elements [6]. Additionally, some aaRSs contain a helix-turn-helix motif (WHEP domain), which is thought to function in protein-protein interactions between synthetases as well as high-affinity interactions with the tRNA [7]. In humans, protein-protein interactions occur between certain cytoplasmic aaRSs that form a large 11-subunit multisynthetase complex (MSC) composed of 8 aaRSs and 3 aaRS-interacting multifunctional proteins (AIMPs), which increases protein synthesis efficiency and is important for other non-canonical aaRS functions [8]. The specificity of each aaRS towards its cognate amino acid and tRNA gives tRNA-charging its necessary high fidelity, however, this is not the only mechanism, as some aaRSs have additional proofreading capabilities carried out in their editing domain. Editing domains are responsible for cleaving incorrectly paired aminoacyl-tRNA molecules to revert misaminoacylation and prevent mistranslation [5].

## 2. Translational Fidelity Guards the Genetic Code

Mutations in an aaRS that cause disease phenotypes can be broadly summarized as loss-of-function and gain-of-function mechanisms. Loss-of-function describes mutations leading to a significant decrease in the original activity of the aaRS. Examples of loss-of-function include mutations in aaRS signal peptides that cause defects in localization; mutations of residues that result in improper folding or dimerization of the aaRS; as well as mutations that impair ATP, tRNA, or amino acid binding and subsequent loss of aminoacylation activity or tRNA release [9]. These mutations lead to a decrease or elimination of the catalytic activity of the aaRS, reduced translational fidelity, and impaired cell growth.

Gain-of-function describes mutations leading to increased or error-prone enzyme activity. Gain-of-function mutations in aaRSs can cause aminoacylation defects, allowing for the ligation of the non-cognate amino acid to the cognate tRNA or the aminoacylation of a cognate amino acid to a non-cognate tRNA [5]. As a result, misaminoacylated tRNA is produced, and the mRNA message is mistranslated to produce mutated proteins. Mistranslation can lead to protein misfolding, aggregation, and a defective proteome [10]. Surprisingly, recent studies have shown that eukaryotic systems can withstand a significant level of mistranslation caused by single nucleotide variants in tRNA molecules. In fact, the abundance of highly tolerable mistranslating tRNAs in the human population indicates that their contribution to disease might be coincidental with other cellular defects [10,11].

Genes encoding aaRSs are essential and play an indispensable role in protein synthesis. Thus, disease-causing mutations in aaRS have become of increasing interest [12]. The aaRS genes are encoded in the human nuclear genome, including 18 aaRS with functions localized to the cytoplasm only, 17 aaRS localized to mitochondria only, and 2 aaRS that function in both the cytoplasm and mitochondria. To date, 56 genetic diseases have been associated with unique mutations in aaRS proteins [13]. The causative role of aaRS mutations in rare human diseases emphasizes the significance and criticality of the aminoacylation step by aaRS enzymes in maintaining translation fidelity and protein homeostasis [6].

## 3. Human Histidyl-tRNA Synthetase

**Figure 2:**
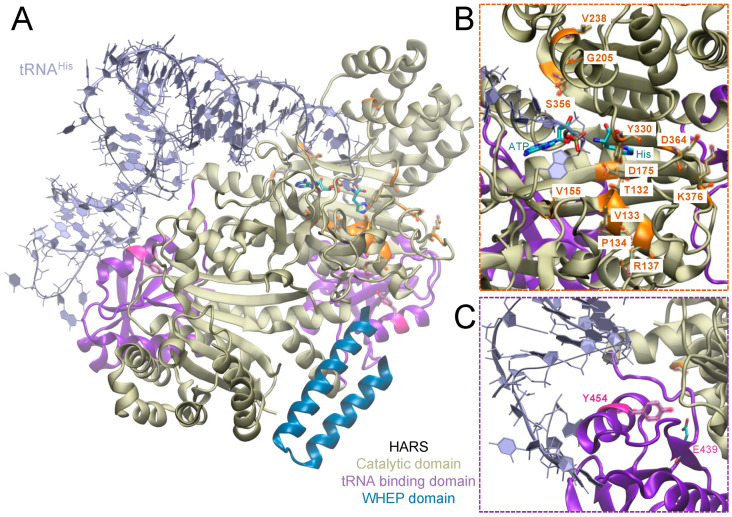
Localization of CMT and USH3B causing HARS mutations. (**A**) Crystal structure of human HARS with CMT and USH3B-causing mutations. The human HARS dimer is shown (pdb:6O76) [29] with the WHEP domain in blue, catalytic domain in tan, and RNA binding domain in purple. The structure was superimposed with the crystal structure from *Thermus thermophilus* (pdb:4RDX) [30], and the tRNA (lavender) is shown. Histidine and ATP are highlighted in the active site. Magnification of (**B**) the catalytic domain with CMT causing mutations depicted in orange, and (**C**) the RNA binding domain with the USH3B mutation Y454S in magenta. The amino acid E439 forms a salt bridge with Y454 that is disrupted in a Y454S mutation. The structure was illustrated using VMD [31].

The human cytosolic histidyl-tRNA synthetase (HARS, HisRS) is a homodimeric class II aminoacyl-tRNA synthetase. *HARS* is a 13-exon, 17.4 kb gene located on chromosome 5 [14,15] and encodes 509 amino acids in a 57.4 kDa polypeptide. HARS consists of an N-terminal WHEP domain, a catalytic domain, and a C-terminal tRNA binding domain (Figure 2) [16]. HARS ligates tRNA^His^ to its cognate amino acid, histidine, producing His-tRNA^His^, which is recruited for the synthesis of cellular proteins. Besides the essential function of HARS in aminoacylation, a non-canonical function of HARS is associated with cell proliferation. Knockdown of HARS in a zebrafish model resulted in increased cell death and reduced proliferation of retinal progenitor cells, suggesting a link between HARS and neuronal progenitor cell survival and proliferation during early development [17]. Furthermore, a splice variant of HARS (HARS∆CD), which lacks the catalytic domain and thus has no aminoacylation function, was found circulating in the bloodstream of patients positive for anti-Jo1 syndrome, an anti-synthetase autoimmune disease. Epitopes in the WHEP domain in particular were targeted by anti-Jo antibodies [18,19].

**Figure 3:**
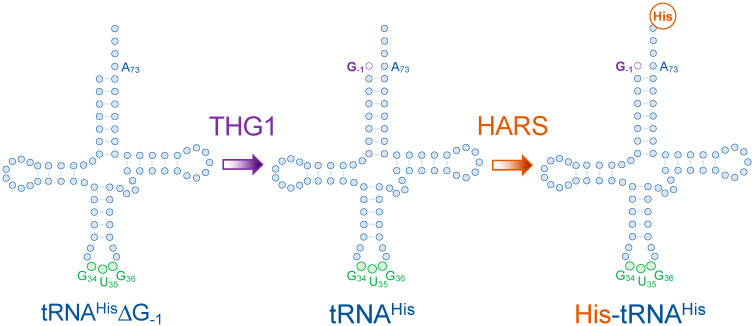
The post-transcriptional addition of the tRNA^His^ identity element (G_-1_) by tRNA^His^ guanylyl transferase (Thg1). Processing of the 5′ end of tRNA^His^ by RNase P leads to the formation of tRNA^His^∆G_-1_. tRNA^His^ guanylyl transferase (Thg1) inserts a guanylyl residue at position −1, opposing to A_73_ in eukaryotes to form mature tRNA^His^. G_-1_ is the identity element in which histidyl tRNA synthetase (HARS) recognizes tRNA^His^ for aminoacylation to form His-tRNA^His^.

The identity element of tRNA^His^ is quite unique, as it involves an additional protein factor that aids in the production of mature tRNA^His^. Eukaryotic tRNA^His^ undergoes post-transcriptional modifications that lead to the addition of its primary identity element: a guanylate residue at position -1 [20,21,22]. Eukaryotic pre-tRNA^His^ lacks this residue as it is not encoded in the genome (tRNA^His^∆G_-1_) [20,21,23,24,25]. Ribonuclease P (RNase P) processes the 5′- end of pre-tRNA^His^ by cleaving at position +1. Then, tRNA^His^ guanylyl transferase (Thg1) adds the guanylate residue at position -1, through its 3′ to 5′ RNA polymerization function, providing mature tRNA^His^ with its primary G_-1_ identity element (Figure 3). Thg1 discriminates between tRNA^His^ and other tRNA molecules by recognizing its anticodon and the discriminator base N_73_. In some bacteria and archaea, G_-1_ is paired with a C_73_ in the acceptor stem of tRNA^His^ but is mispaired with an A_73_ in eukaryotes [21]. Eventually, most bacterial, yeast, or human HARSs recognize the G_-1_ residue, which marks tRNA^His^ for the subsequent histidylation (Figure 3) [26,27,28].

## 4. Disease-Causing *HARS* Mutations

While the aminoacylation and tRNA recognition in bacterial HARS have been studied in detail [32,33,34,35,36], relatively few studies have focused on the functionality and pathogenicity of the human enzyme [37]. Several *HARS* gene mutations have been reported to date as disease-causing (Figure 2, Table 1). Most of these mutations fall within the HARS catalytic domain and are associated with an autosomal dominant neurological disorder, Charcot-Marie-Tooth disease (CMT-type 2W) (Figure 2B) [38,39,40,41,42]. Interestingly, a single missense substitution, Tyr454Ser (Y454S), in the tRNA binding domain of HARS was found to cause Usher syndrome type IIIB (Figure 2C) [43].

Disease-causing mutations in aaRSs can lead to a loss of function, impaired aaRS stability, and/or reduced aminoacylation activity. Gain-of-function mutations can lead to new activities with off-target tRNAs or amino acids, causing misaminoacylation of the cognate tRNA (Figure 1B). Because the ribosome cannot distinguish correctly charged tRNAs from mischarged tRNAs [44], misaminoacylation leads to subsequent mistranslation across the proteome [10,45]. Mistranslation caused by mutations in tRNAs [10,21,46,47,48,49], aaRSs [46], or other components of the protein quality machinery, is increasingly associated with neurodegenerative diseases [10,50], including their ability to genetically modify causative alleles for protein aggregation in Huntington’s disease [48]. Of note, human disease-causing aaRS mutants usually retain a level of activity that is sufficient to sustain the essential functions of protein synthesis [13]. To our knowledge, no disease-causing aaRS mutants with a complete loss of function have been described. Since most of the associated autosomal dominant aaRS diseases affect neuronal activity [13], it is conceivable that dominant gain-of-function mutations and mistranslation might predominantly affect neuronal cells, such as motor neurons in CMT, but the specific CMT-causing mechanism for the aaRS mutants remains to be elucidated.

### 4.1. aaRS Mutations in Charcot Marie Tooth Disease

More than 150 genes are linked to CMT, with at least 59 known disease-causing alleles occurring in six different aaRSs (Table 2). CMT is a heterogeneous genetic disorder that leads to progressive chronic neuropathy affecting the motor and sensory nerves. CMT is the most common inherited neurological disease, affecting 1 in 2500 people. The onset of disease typically occurs in the first or second decade of life [54]. Several types of CMT can be distinguished using electrophysiological studies [55]. The two major CMT types are CMT1 and CMT2. CMT1 causes demyelination and is characterized by slower median or ulnar nerve conduction velocity (NCV), which measures how fast an electrical impulse moves through the nerves. CMT2 is characterized by normal NCV but direct damage to the nerve axon, resulting in reduced muscle action potential, which is the first step of events leading to muscle contraction [55].

All types of CMT present with similar classical clinical features, which include weakness, disappearance of muscle stretch reflexes starting distally from the feet and ascending through the body, as well as bone deformities [56]. Additionally, loss of sensation to pain, temperature, or vibration in the legs often occurs [57]. Patterns of genetic inheritance for CMT include autosomal dominant, autosomal recessive, and X-linked; however, a portion of mutations arise de novo [58]. The 59 mutations in six different aaRSs associated with CMT peripheral neuropathy [59] are found in glycyl-[60], tyrosyl-[61], alanyl-[62], histidyl-[39], methionyl-[63], and tryptophanyl-tRNA [29] synthetases (GARS, YARS, AARS, HARS, MARS, and WARS) (Table 2). CMT-associated aaRSs are mostly dimeric and are not associated with the MSC [64]. MARS presents the exception as being both monomeric and MSC associated, but no CMT mutations are thought to be relevant to MSC complex formation.

Recent data showed that both loss-of-function and gain-of-function of different aaRSs can lead to CMT phenotypes, and both mechanisms may not be mutually exclusive. Loss-of-function in aaRSs may arise from the failure of the enzyme to dimerize or mutations impairing ATP, tRNA, or amino acid binding [7]. A loss of dimerization inhibits the aaRSs from forming functional complexes, resulting in a reduction in aminoacylation activity. Loss of catalytic activity along with impairment of tRNA or amino acid binding may lead to reduced translational fidelity, stalled protein synthesis, and impaired cell growth.

*HARS* mutations V155G, Y330C, and S356N are associated with CMT2W and show a reduction in aminoacylation activity. Interestingly, all three were found to form stable dimers [34]. Mutations T132I, P134H, D175E, and D364Y are thought to induce a conformation-derived gain-of-function, rather than a loss of aminoacylation phenotype [29]. Finally, V133F, V155G, Y330C, and S356N are associated with mistranslation and insoluble protein aggregation, as we demonstrated in a novel yeast model for HARS disease [51]. The gain-of-function mutations in aaRSs are thought to be caused by aminoacylation defects, which allow for an incorrect amino acid to be ligated to a tRNA^His^, leading to mistranslation [3]. These mutations may also result in a loss of specificity for binding of the cognate tRNA. The resulting misacylated tRNA produced by these aaRS is still recognized at the ribosome for protein synthesis as the codon-amino acid pairing is not impaired, and the mRNA message is translated into a mutated protein. Mistranslation resulting from CMT-causing aaRS alleles has a strong potential to cause proteome-wide errors leading to dysfunctional, misfolded, and aggregated proteins that contribute to disease. Not surprisingly, we recently showed the mistranslation of histidine to glutamine and threonine by CMT-causing HARS variants V155G and S356N. Misincorporation of these amino acids was demonstrated by mass spectrometry and was shown to cause a perturbation of the proteome due to threonine and glutamine incorporation at histidine codons [51].

### 4.2. Usher Syndrome Type IIIB

Usher syndrome is an autosomal recessive disease that is associated with severely affected hearing, sight, and balance. This disease has been classified into three categories (Usher syndrome (USH) types I, II, and III) based on the age of onset, severity, and progression of sensorineural hearing loss (SNHL), vision loss due to retinitis pigmentosa (RP), and vestibular dysfunction affecting balance [65,66].

USH-type I is the most severe and is characterized by profound congenital hearing impairment and vestibular dysfunction since birth, and first-decade RP. Usher syndrome is genetically heterogeneous. To date, a total of 14 genes have been associated with Usher syndrome (Table 3) [67]. Six USH1 genes have been identified in proteins that play a role in the development and maintenance of cochlear hair cell bundles. Of these, Harmonin (USH1C), Usher syndrome type-1G protein (USH1G), Cadherin-related 23 (CDH23), Myosin-VIIa (MYO7A/USH1B), and potentially Protocadherin-15 (PCDH15) are thought to form a functional network that mediates mechano-transduction in cochlear hair cells [68]. In a separate pathway to disease, Calcium and integrin-binding protein 2 (CIB2) is expressed in cochlear cells, and mutations in CIB2 are associated with USH1J [69]. USH-type II is the most common among Usher syndrome patients and presents with moderate to severe hearing loss, adolescent-onset RP, and normal vestibular function. USH2 is caused by mutations in three different proteins, including Usherin (USH2A), Adhesion G protein-coupled receptor V1 (GPR98/USH2C/ADGRV1), and Whirlin (DFNB31) [70]. Usherin is thought to play a role in extracellular matrix formation [71], and directly interacts with whirlin and GPR98 to form a protein complex in auditory hair cells and photoreceptor cells [72]. USH-type III is characterized by progressive loss of hearing in the first decade, RP in the second decade, and variable progressive vestibular dysfunction [66,67].

USH-type III is the rarest among Usher syndrome patients. USH-type III is classified into USH3A (OMIM #276902) and USH3B (OMIM #614504) based on the genetic mutation underlying each subtype. USH3A was associated with the gene CLARIN I (CLRN1) in 1995 [73,74,75]. Clarin-1 is an essential protein in the morphogenesis of auditory hair cells [76], and an intronic Clarin-1 mutation leads to aberrant splicing [77] and disrupted Clarin-1 function. Despite Clarin-1 and HARS being the only two proteins associated with USH3, no functional association between these proteins has been identified to date. The genetic mutation underlying USH3B was first identified in 2012 in the Plains populations of Pennsylvania and an Old Order Amish family in Ontario, Canada [43]. A single missense mutation in *HARS* [c.1361A > C], which is inherited in an autosomal recessive manner, leads to a Y454S substitution in patients and is causative for USH3B [43]. Infants with USH3B exhibit normal growth during infancy but experience progressive loss of hearing and sight in the first two decades of life. Rapid clinical deteriorations often occur in response to febrile illnesses. Most patients experience episodic hallucinations due to acute viral infections [43].

HARS Y454S is likely the best clinically characterized disease-causing mutation in human HARS [43,51,53], with a dedicated clinical trial nearing completion (Clinical Trial ID: NCT02924935). The crystal structures of human HARS show that Y454S is located in the interface between the tRNA binding domain and the catalytic domain of the opposing monomer (Figure 2C), suggesting that it may alter anticodon recognition or catalytic activity [30,78]. The substitution of tyrosine to serine at 454 creates a gap between Glu439 and Ser454, destabilizing the hydrogen bonding interaction between both residues (Figure 2C) [43]. Consequently, it was expected for Y454S to influence tRNA binding, aminoacylation, and perhaps the dimerization of the enzyme. A study on murine IMCD3 cells showed, however, only a minor decrease in aminoacylation by mutant murine HARS and unaffected localization and dimerization with endogenous wild-type HARS [43]. Furthermore, a recent study on USH3B patient fibroblasts has shown that the Y454S mutation does not alter the binding specificity to tRNA^His^, nor does it affect the overall catalytic activity of HARS. On the other hand, recombinant Y454S HARS protein was shown to be thermolabile due to a significant decrease in melting temperature when compared to wild-type HARS, as well as its failure in incorporating histidine into the proteome of patients’ fibroblasts at elevated temperatures (43 °C) [53]. It is therefore likely that HARS Y454S leads to HARS destabilization and reduced catalytic activity, but it is also feasible that other functions, including non-canonical functions that lead to circulating HARS fragments in the bloodstream, may be impacted by the Y454S mutation [18,19].

## 5. Pathways to a Cure

Both USH3B and CMT2W are debilitating diseases, yet no current cure is available. Common treatments, such as braces and walking aids, alleviate CMT symptoms, but neither prevent disease onset nor halt or reverse disease progression. For USH3B, amino acid supplementation as a treatment is currently being considered in a clinical trial, and with the astonishing progress in RNA therapeutic technology, treatment of patients with tRNA supplements may also be possible in the near future.

Several non-canonical functions of GARS and KARS are associated with neurodegenerative disorders [79]. These functions and aaRS as therapeutic targets were recently reviewed in detail [5]. Another aaRS-related disease, anti-synthetase syndrome, is a rare autoimmune disorder where the production of antibodies against aaRS leads to myositis, interstitial lung disease, or arthritis [80]. Auto-antibodies against eight aminoacyl-tRNA synthetases have been associated with the clinical manifestation of anti-synthetase syndrome, with the Jo-1 antibody, which is specific for HARS, being the most frequent one [80]. No disease-specific treatment for anti-synthetase syndrome is currently available, and the 5-year survival rate of anti-Jo syndrome with immunosuppressive drugs is estimated at approximately 90% [80].

Recent advances in the treatment of CMT and other aaRS-linked diseases have focused on amino acid supplementation. Several studies show that prolonged, high doses of alanine [81], histidine [82], and glycine [83] supplementation are regarded as safe. Excitingly, and as mentioned above, histidine supplementation is being explored as a treatment option for USH3B patients in a clinical trial. Histidine, like many amino acids, is a readily available supplement in health food stores at a low cost. We found that mistranslation or HARS deficiency caused by CMT-associated aaRS mutations can be alleviated by histidine or tRNA^His^ supplementation in vitro [51]. For example, in a yeast model of CMT2W with the HARS mutations V133F, V155G, Y330C, or S356N, histidine supplementation rescued the growth defect and prevented insoluble protein accumulation in two of these mutants (V155G and S356N), indicating that histidine supplementation may be a viable treatment option for these patients [51].

While HARS V155G and S356N were rescued by histidine supplementation, the same treatment increased the toxicity of human HARS V1333F and Y330C mutant alleles in yeast cells, indicating that histidine is not an adequate treatment option and other avenues need to be explored. Several CMT-associated mutations in GARS and MARS are localized in the tRNA binding sites of the proteins (Table 1) and may affect tRNA binding. Some mutants affect amino acid specificity or aminoacylation efficiency, whereas other mutants lead to altered tRNA binding to the aaRS, which may be causative of a CMT phenotype. Interestingly, tRNA overexpression compensates for increased tRNA binding by a CMT-GARS mutant in mouse models [84]. In this case, mutations near the GARS tRNA binding domains resulted in altered tRNA specificity or binding, which was compensated for by overexpression of cognate tRNAs [84]. In several recent breakthrough studies, tRNA delivery was used to treat cystic fibrosis [85], mucopolysaccharidosis type I [86], and CMT [84] in mouse models. With recent advances in RNA delivery [87], tRNA medicine is a promising approach to curing CMT-causing phenotypes and will enable next-generation personalized treatments tailored to an individual’s particular CMT-causing allele.

## Figures and Tables

**Table 1:** Disease-causing mutations in human *HARS*.

*HARS* Mutation	Publication
**T132I**	Safka Brozkova et al., 2015 [39], Blocquel et al., 2019 [29]
**T132S**	Safka Brozkova et al., 2015 [39]
**V133F**	Royer-Bertrand et al., 2019 [40], Qiu et al., 2022 [51]
**P134H**	Safka Brozkova et al., 2015 [39], Blocquel et al., 2019 [29]
**R137Q**	Vester et al., 2013 [38], Mullen et al., 2021 [52]
**V155G**	Abbott et al., 2018 [41], Mullen et al., 2021 [52], Qiu et al., 2022 [51]
**D175E**	Safka Brozkova et al., 2015 [39], Blocquel et al., 2019 [29]
**G205D**	Vester et al., 2013 [38]
**V238A**	Vester et al., 2013 [38]
**Y330C**	Abbott et al., 2018 [41], Mullen et al., 2021 [52], Qiu et al., 2022 [51]
**S356N**	Abbott et al., 2018 [41], Qiu et al., 2022 [51]
**D364Y**	Safka Brozkova et al., 2015 [39], Blocquel et al., 2019 [29]
**K376R**	Vester et al., 2013 [38]
**Y454S**	Puffenberger et al., 2012 [43], Abbott et al., 2017 [53], Qiu et al., 2022 [51]
**P505S**	Vester et al., 2013 [38]

**Table 2:** CMT disease-associated mutations in aaRSs.

	Active Site	tRNA Binding Domain	Other Domains
**AARS**	N71Y, G102R, R326W, R329H, E337K		S627L, E688G, E778A, D893N
**GARS**	E71G, L129P, D167Y, D146N, C157R, H162R, C201R, S211F, H216R, L218Q, P224L P234K/Y, M238R, G240R, P244L, S265Y, S273R, E279D, I280F, G327R, P336H/R, H418R, H472R, D500N, K510Q, G526R	M555V, S581L, G625R	K27R, K27P, A57V
**HARS**	T132I, P134H, R137Q, V155G, D175E, Y330C, S356N, D364Y	Y454S (USH3B)	
**MARS**	A397T	R618C, R737W, P800T	
**YARS**	G41R, D81I, D(153–156), E196K, E196Q		
**WARS**	H257R, D314G		

**Table 3:** Genes and loci associated with Usher syndrome. Loci are denoted in italics.

**Associated genes/*loci***	**USH-Type I**	**USH-Type II**	**USH-Type III**
	MYO7A/USH1B	USH2A	CLRN1
	CDH23/USH1D	GPR98/USH2C	**HARS**
	PCDH15	DFNB31/USH2D	
	USH1C		
	*USH1E*		
	USH1G		
	*USH1H*		
	*USH1K*		
	CIB2		

## Data Availability

Data sharing not applicable.
